# IDH1 mutation is detectable in plasma cell-free DNA and is associated with survival outcome in glioma patients

**DOI:** 10.1186/s12885-023-11726-0

**Published:** 2024-01-03

**Authors:** Stefania Crucitta, Francesco Pasqualetti, Alessandra Gonnelli, Martina Ruglioni, Giovanna Irene Luculli, Martina Cantarella, Valerio Ortenzi, Cristian Scatena, Fabiola Paiar, Antonio Giuseppe Naccarato, Romano Danesi, Marzia Del Re

**Affiliations:** 1 Unit of Clinical Pharmacology and Pharmacogenetics, Department of Clinical and Experimental Medicine, University of Pisa, Pisa, Italy; 2 Radiation Oncology, Department of Medicine and Oncology, University of Pisa, Pisa, Italy; 3 Department of Oncology, University of Oxford, Oxford, UK; 4 Division of Pathology, Department of Translational Research & New Technologies in Medicine & Surgery, University of Pisa, Pisa, Italy; 5 Department of Oncology and Hemato-Oncology, University of Milano, Via Festa del Perdono, 7, Milano, 20122 Italy

**Keywords:** Liquid biopsy, cfDNA, IDH1, Glioma

## Abstract

**Background:**

Circulating cell-free DNA (cfDNA, liquid biopsy) is a powerful tool to detect molecular alterations. However, depending on tumor characteristics, biology and anatomic localization, cfDNA detection and analysis may be challenging. Gliomas are enclosed into an anatomic sanctuary, which obstacles the release of cfDNA into the peripheral blood. Therefore, the advantages of using liquid biopsy for brain tumors is still to be confirmed. The present study evaluates the ability of liquid biopsy to detect IDH1 mutations and its correlation with survival and clinical characteristics of glioma patients.

**Methods:**

Blood samples obtained from glioma patients were collected after surgery prior to the adjuvant therapy. cfDNA was extracted from plasma and IDH1 p.R132H mutation analysis was performed on a digital droplet PCR. χ2-test and Cohen k were used to assess the correlation between plasma and tissue IDH1 status, while Kaplan Meier curve and Cox regression analysis were applied to survival analysis. Statistical calculations were performed by MedCalc and GraphPad Prism software.

**Results:**

A total of 67 samples were collected. A concordance between IDH1 status in tissue and in plasma was found (*p* = 0.0024), and the presence of the IDH1 mutation both in tissue (138.8 months vs 24.4, *p* < 0.0001) and cfDNA (116.3 months vs 35.8, *p* = 0.016) was associated with longer median OS. A significant association between IDH1 mutation both in tissue and cfDNA, age, tumor grade and OS was demonstrated by univariate Cox regression analysis. No statistically significant association between IDH1 mutation and tumor grade was found (*p* = 0.10).

**Conclusions:**

The present study demonstrates that liquid biopsy may be used in brain tumors to detect IDH1 mutation which represents an important prognostic biomarker in patients with different types of gliomas, being associated to OS.

## Background

Liquid biopsy recently emerged as a new approach to investigate the molecular profile of solid tumors by detecting gene alterations and obtaining potential prognostic and predictive biomarkers across different cancers [1–3]. Liquid biopsy has gained interest due to its advantages being a minimally invasive, sensitive, repeatable, and feasible alternative to tissue biopsy, having the ability to capture heterogeneity across multiple areas of tumors [2, 4–6]. The term “liquid biopsy” is mainly referred to the analysis of circulating free DNA (cfDNA) extracted from plasma, which contains a small amount of tumor DNA (ctDNA); however, this concept is also applied to different biological fluids such as blood, cerebrospinal fluid (CSF), urine, saliva, and to several analytes such as cell-free RNA, circulating tumor cells, extracellular vesicles (EVs), RNA and non-coding miRNA [7–13]. Nowadays, several national and international recommendations are available, regarding the use of liquid biopsy in clinical practice, which mainly refer to the use of plasma cfDNA, and suggest the use of alternative sources only in specific clinical trials and research studies [14, 15]. However, when using liquid biopsy, one of the major challenge is represented by the false negative results, mainly related to anatomic barriers and disease biological characteristics [16, 17]. While cfDNA has been detected in several types of cancers including breast, lung, pancreatic, melanoma and colorectal cancer [18–27], few studies have been able to identify cfDNA in peripheral blood in patients with glioma, due to cfDNA difficulties of crossing the blood–brain barrier (BBB), and since its release can change depending on histopathology, localization, and tumor grade [17, 28–32].

Ideally, the use of the liquid source close to the disease site would be the optimal solution; therefore, the CSF-derived cfDNA would be the best source of biological material to study the molecular profile of brain tumors through liquid biopsy [33–38]. Published studies using sensitive methods, such as digital PCR, demonstrated that higher concentrations of cfDNA may be found in CSF compared to plasma, suggesting that cfDNA in CSF could be used as a molecular marker to identify mutations and longitudinally monitor changes in brain tumors [39–42]. However, CSF is an invasive, complex, and uncomfortable approach, requiring specific expertise and associated with additional risks following its repeated use [34].

Among brain tumors, high-grade gliomas are considered the most common malignancies in adults and their classification has traditionally been based on histopathological findings supplemented by tissue-based tests, grade, and genomic alterations [43–46]. In particular, the presence of the IDH1 p.R132H amino acid substitution in tissue has been found to be the most common subtype with a 90% of prevalence among IDH1-mutant tumors [47–50]. Different studies suggested that IDH-mutant gliomas have a significantly improved prognosis, independently of age and grade, as compared to IDH-wild-type tumors [51–54]. Accordingly, the World Health Organization (WHO) classification of diffuse gliomas under the 2021 update has been dependent largely on IDH1 mutation status together with 1p/19q-codeletion [55–57]. In particular, the new classification includes astrocytoma, IDH-mutant (Grade 2, 3, 4), oligodendroglioma, IDH-mutant, and 1p/19q-codeleted (Grade 2, 3) and GBM, IDH-wildtype (Grade 4) [57].

Therefore, the use of IDH1 mutation status as a prognostic biomarker of tumor molecular evolution has allowed easier and more accurate risk stratification of glioma patients and could be a key tool to improve their personalization of treatments. Moreover, IDH-1 and -2 recently became a predictive biomarker of response to specific IDH inhibitors, such as vorasidenib for low grade glioma [58].

In the present study, we assessed the feasibility of detecting plasma-cfDNA IDH1 mutation in gliomas and the results were correlated with survival and clinical characteristics of patients affected by gliomas.

## Methods

### Patients and data collection

The present retrospective pharmacogenetic study included patients with glioma referred to the University Hospital of Pisa (Italy) from 2016 to 2019.

Patients were selected according to the following criteria: histologic neuronal-glial tumors diagnosis according to the fourth edition of the WHO classification (2016); IDH1 status previously determined by Sanger sequencing of tumor DNA as per standard laboratory procedures; clinical data and follow-up available in the neuro-oncology database; and written informed consent before enrollment in the study [57].

Blood samples were collected after surgery prior to the adjuvant therapy. The study has been approved by the local Human Investigations Committee in accordance with the Declaration of Helsinki (Comitato Etico Area Vasta Nord Ovest Toscana, Prot. Number 560/2015).

### Circulating free DNA extraction and IDH1 mutation analysis

Twelve ml of blood were collected in EDTA tubes and centrifuged for 10 min at 1900×g within 2 h from sampling to collect plasma and stored at − 80 °C until analysis. Plasma samples were centrifuged again for 15 min at 1900×g to remove cellular debris. cfDNA was isolated from 3 ml of plasma using the QIAamp Circulating Nucleic Acid Kit (Qiagen, Valencia, CA). The analysis of IDH1 p.R132H mutation was performed by the QX200 digital droplet PCR (Bio-Rad, Hercules, CA) as previously reported [59].

### Statistical analysis

Categorical variables and patient clinical outcomes were described by absolute and relative frequencies, while quantitative factors such as age by mean ± standard deviation (STD). Overall survival (OS) was measured as the length of time from the diagnosis to death from any cause or last follow-up. To find which IDH genomic factors are related to OS, survival curves were estimated according to the Kaplan-Meier method, and differences between curves were calculated using the log-rank test. Univariate analysis was performed by Cox hazard regression model to evaluate independent risk factors for OS. The associations between IDH1 somatic mutation and the other categorical variables in the sample were assessed using χ2-test. Specificity and sensitivity were also calculated using ROC analysis. Agreement between IDH1 mutation detection in tissue and plasma was calculated with the Cohen κ test. Differences were considered significant at *p* < 0.05. All statistical calculations were performed with MedCalc Statistical Software version 14.8.1 (MedCalc Software bvba, Ostend, Belgium; http://www.medcalc.org; 2014) and GraphPad Prism 9.0.0 (GraphPad Software, San Diego, California USA, www.graphpad.com).

## Results

### Patients’ characteristics

A total of 67 samples from patients affected by a glioma were collected; the average patient age at the time of first blood collection was 54 years old (range 28–84); sex ratio was 0.86 (31 males and 36 females). The majority of patients (64%) presented newly diagnosed tumors, while a subset (3%), comprising 2 cases of oligoastrocytoma and 1 of anaplastic astrocytoma, underwent analysis subsequent to a diagnosis of recurrence and prior to treatment initiation. Histopathological subtypes of brain tumors analyzed according with the fourth edition of the WHO Classification (2016) included oligodendroglioma, diffuse astrocytoma, anaplastic astrocytoma, ganglion astrocytoma, oligo-astrocytoma and glioblastoma (GBM), with the latter being the most frequent diagnosis in our cohort (64.1%). Table 1 reports the clinical characteristics of the cohort analyzed.

**Table 1:** Clinical characteristics of patients

	Total of patients (***n*** = 67)
**Age at the diagnosis, median (range)**	54 (24–84)
**Gender, n (%)**	
Male	31 (46.3)
Female	36 (53.7)
**Astrocytic, oligodendroglial and neuronal-glial tumors, n (%)**	
Oligodendroglioma	3 (4.5)
Diffuse astrocytoma	5 (7.5)
Astrocytoma, anaplastic	10 (14.9)
Ganglion astrocytoma	1 (1.5)
Oligoastrocytoma	5 (7.5)
Glioblastoma	43 (64.1)
**Tumour grade, n (%)**	
Low (I, II)	11 (16.4)
High (III, IV)	56 (83.6)
**Tumor Site, n (%)**	
Frontal	29 (43.3)
Temporal	7 (10.4)
Parietal	9 (13.4)
Occipital	1 (1.5)
Multiple	19 (28.4)
Other	2 (3)
**Type of surgery, n (%)**	
STR	40 (59.7)
GTR	27 (40.3)
**Tissue IDH1 mutational status, n (%)**	
No mutation	46 (68.7)
Mutation	21 (31.3)
**1p19q co-deletion status**	
Co-deletion	6 (8.6)
No co-deletion	58 (86.9)
Unknown	3 (4.5)
**MGMT methylation status**	
Methylated	29 (43.3)
Unmethylated	15 (22.4)
Unknown	23 (34.3)

*Abbreviations: STR* subtotal resection, *GTR* gross total resection

Tissue IDH1 mutational status was available for all patients included in the study; 21 patients (31.3%) were carriers of the IDH1 p.R132H mutation and 46 patients (68.7%) were IDH1 wild type. More specifically, the highest number of patients with IDH1 mutation in tissue was found in the anaplastic astrocytoma group (38.1%), following by diffuse astrocytoma (23.8%), GBM (14.3%), oligodendroglioma (14.3%), and oligoastrocytoma (9.5%).

Twelve out of twenty-one cases (57.2%) with IDH1 mutation were located in the frontal lobe, while 2/21 (9.5%) involved the temporal and parietal lobes, and 7/21 (33.3%) were multifocal. Moreover, 11/21 (52.4%) of patients underwent a subtotal resection (STR), whereas 10/21 (47.6%) patients had a gross total resection (GTR).

Based on the 2021 WHO Classification of Tumors of the Central Nervous System, brain tumors were classified into seven different subgroups [55]. According to this, patients were grouped in GBM (65.6%), oligodendroglioma (4.5%), astrocytoma grade 2 (7.5%), astrocytoma G III (11.9%), astrocytoma G IV (4.5%) and ganglion astrocytoma (1.5%). However, 1p19q co-deletion status was not available for 3 patients (4.5%) identified as oligoastrocytoma in the WHO 2016 classification. For this reason, the 2021 update could not be applied to these patients.

More specifically, the highest number of patients with IDH1 mutation in tissue was found in the astrocytoma grade (G) III (38.1%), following by astrocytoma G II (23.8%), oligodendroglioma (14.3%), astrocytoma G IV (9.5%), and oligoastrocytoma (9.5%) (Table 2).

**Table 2:** Detection of IDH1 mutation in tissue and plasma according to the WHO classification 2016 and 2021 update

**WHO Classification (2016)**	**Distribution of IDH1 p.R132H mutation**				
	*Only Tissue*	*Only Plasma*		*Both Tissue and Plasma*	
	N. samples	N. samples	Median AF	N. samples	Median AF
Oligodendroglioma (*n* = 3)	1	–	–	2	0,36
Diffuse astrocytoma (n = 5)	1	–	–	4	0,21
Anaplastic astrocytoma (*n* = 8)	5	–	–	3	0,20
Oligo-astrocytoma (*n* = 2)^a^	2	–	–	–	–
GBM (n = 8)	2	5	0,16	1	0,12
**WHO Classification (2021)**	**Distribution of IDH1 p.R132H mutation**				
	*Only Tissue*	*Only Plasma*		*Both Tissue and Plasma*	
	N. samples	N. samples	Median AF	N. samples	Median AF
Oligodendroglioma (n = 3)	1	–	–	2	0,36
Astrocytoma G II (n = 5)	1	–	–	4	0,21
Astrocytoma GIII (n = 8)	5	–	–	3	0,20
Astrocytoma G IV (n = 3)	2	–	–	1	0,12
GBM (n = 5)	–	5	0,16	–	–

^a^ No information about 1p19q co-deletion status was available for this subgroup of patients

### Detection of IDH1 p.R132H mutation in plasma cfDNA

**Fig. 1:**
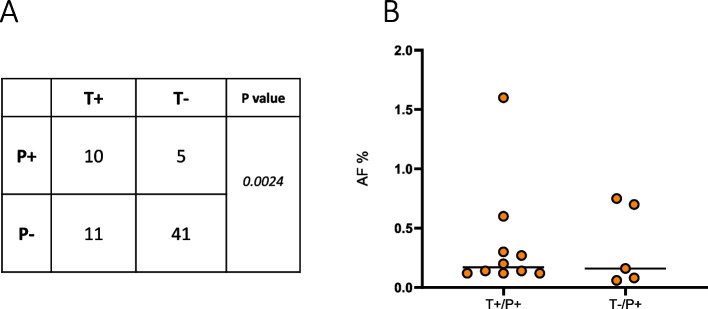
Concordance table for the presence of the IDH1 mutation both in tissue and in plasma (**A**). Mutant amplification ratio expressed as allelic fraction (AF) of IDH1 p.R132H mutation detected by ddPCR (**B**)

Sixty-seven samples were analyzed for IDH1 p.R132H mutation in cfDNA and tissue; in particular, 10 samples (15%) were positive both in tissue and in cfDNA (T+/P+), while five patients (7.3%) were IDH1 wild type in tumor tissue but positive in cfDNA (T−/P+). Finally, 11 patients were found to have IDH1 mutated tumor tissue and no IDH1 mutation in plasma (16.4%) (Fig. 1a). A Chi-squared test was conducted and a statistically significant difference across the two groups was observed (*p* = 0.0024), indicating a potential relationship between the two categorical variables (tissue and plasma). Compared to tissue, the sensitivity and specificity for IDH1 mutation in plasma was 47.6% (CI 25.7–70.2) and 89.1% (CI 76.6–96.4), respectively. Moreover, a fair agreement between IDH1 mutation status detected in tissue and plasma was found, with a Cohen κ of 0.40 (95% CI, 0.16–0.64). Figure 1b reports the IDH1 Fractional Abundance (AF), expressed as percentage, in T+/P+ and T−/P+ group of patients. In particular, for T+/P+ patients, the median AF% was 0.17 (0.12–1.60), whereas for the T−/P+ group, the median AF% was 0.16 (0.06–0.75).

Table 3 summarizes the characteristics of discordant cases (T−/P+, T+/P-). Among T−/P+ group, the blood sample of 1 patient was collected during the follow-up, in concomitance of the progression of the disease.

**Table 3:** Clinical characteristics of IDH1 mutant discordant cases

	T−/P+ group (***n*** = 5)	T+/P- group (***n*** = 11)
**Age at the diagnosis, median (range)**	58 (44–70)	41 (24–73)
**Gender, n (%)**		
Male	2 (40)	7 (63.7)
Female	3 (60)	4 (36.3)
**Astrocytic, oligodendroglial and neuronal-glial tumors, n (%)**		
Oligodendroglioma	–	1 (9.1)
Diffuse astrocytoma	–	1 (9.1)
Astrocytoma, anaplastic	–	5 (45.4)
Ganglion astrocytoma	–	–
Oligoastrocytoma	–	2 (18.2)
Glioblastoma	5 (100)	2 (18.2)
**Tumour grade, n (%)**		
Low (I, II)	–	3 (27.3)
High (III, IV)	5 (100)	8 (72.7)
**Tumor Site, n (%)**		
Frontal	1 (20)	6 (54.6)
Temporal	2 (40)	–
Parietal	1 (20)	–
Occipital	–	–
Multiple	1 (20)	4 (36.3)
Other	–	1 (9.1)
**Type of surgery, n (%)**		
STR	5 (100)	5 (45.4)
GTR	–	6 (54.6)
**Tissue IDH1 mutational status, n (%)**		
No mutation	5 (100)	–
Mutation	–	11 (100)
**1p19q co-deletion status**		
Co-deleted	–	2 (18.2)
Non-co-deleted	5 (100)	8 (72.7)
Unknown	–	1 (9.1)
**MGMT methylation status**		
Methylated	1 (20)	7 (63.7)
Unmethylated	3 (60)	–
Unknown	1 (20)	4 (36.3)

Considering the T+/P+ group (*n* = 10), the highest number of patients with IDH1 mutations in cfDNA were found in the diffuse astrocytoma (40%), following the anaplastic astrocytoma (30%), oligodendroglioma (20%), and GBM (10%) (Table 2). According to the 2021 WHO Classification, the highest number of patients with IDH1 mutation in plasma was found in the astrocytoma G II (40%), and G III (30%), oligodendroglioma (20%), astrocytoma G IV (10%) (Table 2). Six of ten patients (60%) underwent a STR and 4/10 (40%) had a GTR.

Focusing on the T−/P+ group (*n* = 5), all patients presented a GBM high grade tumor (Table 2) involving the temporal lobe (2/5, 40%), frontal lobe (1/5, 20%), parietal lobe (1/5, 20%), or multiple sites (1/5, 20%). Interestingly, all the patients underwent a STR (100%).

**Fig. 2:**
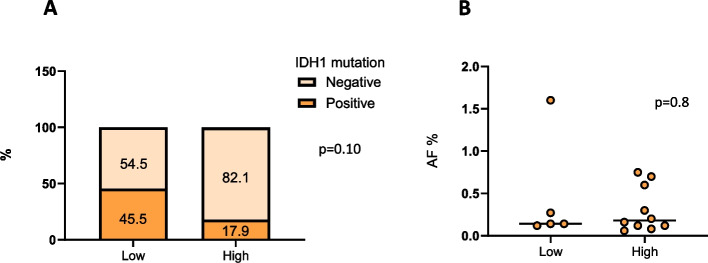
IDH1 mutation frequencies detected in cfDNA according to tumor grade (**A**). IDH1 mutation expressed as allelic fraction (AF) detected in cfDNA according to tumor grade (**B**)

Overall, a correlation analysis of the presence of IDH1 mutation in cfDNA with the histological grade of tumor was also performed (Fig. 2a). Five of 11 low-grade gliomas had the IDH1 p.R132H mutation in tissue and plasma. Among the 56 patients with high-grade glioma, the mutation was detected in the plasma of 5 patients (8.9%) with an IDH1 p.R132H mutant tumor. A Chi-squared test was conducted to evaluate the potential association between tumor grade and the presence of IDH1 mutation in cfDNA. No statistically significant difference across the two examined groups was observed (*p* = 0.10).

Figure 2B reported the correlation analysis of the histological grade with the presence of the IDH1 (AF%) mutation in plasma cfDNA, no statistically significant association was found (*p* = 0.8).

**Fig. 3:**
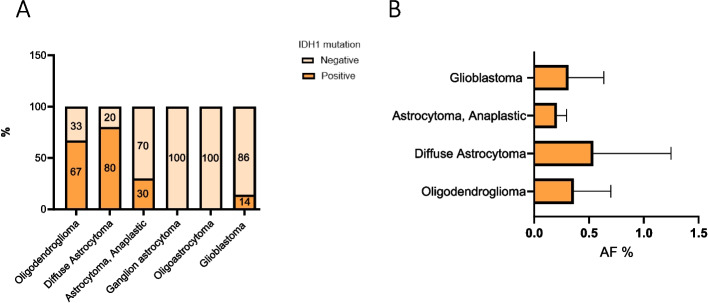
IDH1 mutation frequencies detected in cfDNA according to histological subtype (**A**). IDH1 mutation expressed as allelic fraction (AF) detected in cfDNA according to histological subtype (**B**)

Figure 3 reports the IDH1 alteration rate in plasma (Fig. 3A) and the median AF% (IC 95%) per patient stratified by histologic subtype (Fig. 3B). Diffuse astrocytoma, oligodendroglioma and glioblastoma revealed greater AF% than other subtypes.

### IDH1 mutation in plasma and tissue predicts overall survival in glioma tumors

**Fig. 4:**
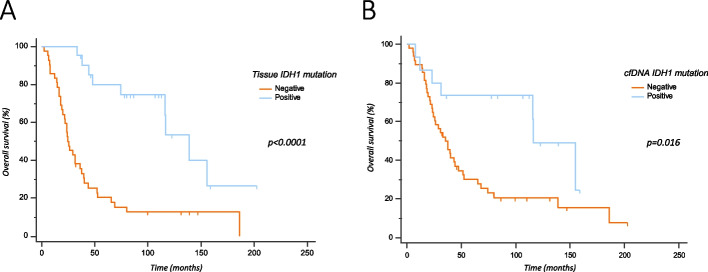
Overall survival of glioma patients stratified as per IDH1 mutational status detected in tissue (**A**) and cfDNA (**B**)

Median overall survival of the entire population was 40.3 months. Considering the patients whose IDH1 status was assessed on tissue, as expected, median OS was significantly longer in patients with IDH1 p.R132H mutation vs IDH wild type (138.8 months vs 24.4; *p* < 0.0001; Fig. 4A). Accordingly, also for patients with the IDH1 assessment on cfDNA, median OS was significantly longer in patients with IDH1 p.R132H mutation vs IDH wild type (116.3 months vs 35.8, *p* = 0.016; Fig. 4B).

We conducted univariate cox regression analysis using clinical variables such as age, gender, tumor grade, type of surgery and IDH1 mutation status finding a statistically significant association between IDH1 mutation both in tissue and cfDNA, age, tumor grade and OS (Table 4). Sample size was unfortunately not large enough to allow a multivariate analysis.

**Table 4:** Univariate analysis for overall survival

Variables	HR (95% CI)	***p***-value
***Age (years)***		
*≤54*	Reference	
*> 54*	2.83 (1.51–5.33)	***0.0013***
***Gender***		
*Male*	Reference	
*Female*	0.82 (0.46–1.45)	0.49
***Tumour grade***		
*Low (I, II)*	Reference	
*High (III, IV)*	5.54 (1.68–18.26)	***0.005***
***Type of Surgery***		
*STR*	Reference	
*GTR*	0.98 (0.54–1.80)	0.97
***Tissue IDH1 mutation status***		
*No mutation*	Reference	
*Mutation*	0.22 (0.10–0.45)	***0.0001***
***Plasma cfDNA IDH1 mutation status***		
*No mutation*	Reference	

*Abbreviations: STR* subtotal resection, *GTR* gross total resection, *CI* confidence interval, *HR* hazard ratio

Considering patients with wild-type IDH1 status in tissue but a mutant IDH1 status in plasma (*n* = 5), median OS was 65.8 months. Despite a subgroup analysis was not performed due to the limited sample size, the longer PFS may confirm the presence of the IDH1 mutation, or the presence of a higher level of tumor molecular heterogeneity.

## Discussion

Our study examined the feasibility of using liquid biopsy for patients affected by a brain tumor, and the association between the mutational status of IDH1 in plasma, survival outcomes and clinical characteristics of a cohort of glioma patients. In our study, a statistically significant concordance between IDH1 mutation detection in tissue and cfDNA was found (*p* = 0.0024), suggesting that liquid biopsy is a technique capable of integrating tissue biopsy, especially in elderly patients or patients with tumors close to critical areas. In particular, IDH1 mutation was detected in the cfDNA of 10 out of 21 patients harboring an IDH1 mutant tumor, and in 5 patients with IDH1 wild type tumor tissue (*n* = 41), suggesting that liquid biopsy may support tissue biopsy in the detection of IDH1 mutation. Similarly, previous studies were able to identify the IDH1 mutation using a digital PCR technology in the plasma of 50–60% of patients with IDH-mutant gliomas [29, 30]. Interestingly, all patients with IDH1 wild type tumor but with detectable IDH1 mutation in cfDNA had a subtotal resection, and a residual tumor burden was still present in the brain. Therefore, a shedding of cfDNA in the circulation could be expected as a consequence of this type of surgery [60, 61]. For this reason, our analysis could more accurately represent the entire heterogeneity of the tumor compared to tissue biopsy, explaining the detection of the IDH1 mutation in the cfDNA of a subgroup of patients negative on tissue. On the other hand, in our cohort eleven patients presented IDH1 mutated tumor tissue and no IDH1 mutation in plasma, suggesting that the blood-brain barrier could limit the release of cfDNA and consequently, the detection of IDH1 mutation. Recently, a meta-analysis highlighted the complexity of cfDNA analysis to detect the mutational status in glioma patients [28]. cfDNA analysis resulted to have high specificity (0.98; 95% CI 0.96–0.99) but a relatively moderate sensitivity (0.69; 95% CI 0.66–0.73) and a high grade of heterogeneity (I2 = 73.1%, *p* < 0.05) of sample source and assay methods [28]. These latter could affect the sensitivity of the cfDNA and the accuracy of the findings, suggesting that cfDNA could be used only as an auxiliary tool for molecular assessment of glioma [62].

In our study, patients were divided into different groups depending on the brain tumor’s histopathological subtypes and tumor grade, finding a higher rate of IDH1 mutation (AF%) in the diffuse astrocytoma, glioblastoma and oligodendroglioma. However, the association between tumor grade and the presence of IDH1 mutation (*p* = 0.10) was not statistically significant. Specifically, the majority of patients with a low-grade brain tumor correlated with the presence of the p.R132H mutation, while the high-grade group, which counted about 82% of patients, did not show IDH1 mutation. This is in accordance with previous studies, which reported that IDH1 mutation occurs in the majority of low-grade gliomas and less frequently in high-grade gliomas [50, 63, 64]. Accordingly, Yan et al. confirmed that IDH1 mutations are more frequent in G II–III astrocytomas and oligodendrogliomas and less frequently in GBM, underlining the correlation between the presence of IDH1 mutation and tumor grade [63].

To date, few data have reported the association between IDH1 mutations with tumor localization [65]. In the present study, we found that the majority of gliomas (57.2%) with IDH1 mutations were located in the frontal lobe, followed by gliomas affecting multiple sites (33.3%) and temporal or parietal tumors (9.5%), suggesting a potential relationship between IDH1 mutations and tumor localization.

Interestingly, our study evaluated the role of the IDH1 mutation detected in tissue and plasma and the survival outcome. Patients carrying IDH1 mutation p.R132H have a higher survival rate than patients not carrying the mutation. Accordingly, several studies highlighted the positive correlation between IDH1 p.R132H mutation and survival of patients with gliomas [66–68]. Sanson et al. highlighted that the IDH1 codon 132 mutation is associated with the genomic profile of the tumor and constitutes an independent prognostic marker in G II to IV gliomas (*p* = 0.00021) [66]. Similarly, Polivka et al. affirmed that patients with IDH1 p.R132H mutation had a significantly longer OS than patients with wild-type IDH1 (270 versus 130 days; *p* < 0.024) [67].

A survival rate of 65.8 months was found in patients with a wild type IDH1 status in tissue but a mutant IDH1 status in plasma (T−/P+), which is approximately twice as long than the median OS of tissue (24.4 months) or plasma (35.8 months) IDH1 wild type patients of our cohort, assuming the real presence of the IDH1 mutation. However, due to the small sample size, appropriate statistical analyses could not be performed.

In our study, the evaluation of the O-6-methylguanine-DNA methyltransferase (MGMT) status was not available for all patients. In this context, different studies suggested the importance of combined IDH1 and MGMT analysis to predict survival in patients with different types of gliomas [65, 69]. Patients harboring IDH1 mutation and MGMT methylation had the more favorable outcomes, followed by patients with IDH1 mutation and unmethylated MGMT promoter, and patients without IDH1 mutation and unmethylated MGMT promoter [69]. There is compelling evidence that liquid biopsy, especially using cfDNA, offers an accurate and accessible approach to capture the landscape of brain tumor-related molecular alterations, allowing diagnosis and characterization of glioma patients [35, 38]. Liquid biopsy is already used in several tumors, especially for treatment monitoring, and recent several studies reported its usefulness in the management of glioma patients [38, 70–73]. Moreover, liquid biopsy has found applicability as potential minimally invasive alternative to traditional tissue surgery, especially in difficult scenarios (i.e unavailable tissue, poor clinical conditions of patients), bypassing its spatial and temporal biases, and for the research of diagnostic and prognostic biomarkers, enabling clinicians to improve the neuro-oncology traditional monitoring of glioma patients [32, 33, 35–37, 74–76]. Moreover, the assessment of the IDH-1 and -2 status will become crucial since vorasidenib, a new oral brain-penetrant IDH-1/2 inhibitor inhibitor, showed linical activity in IDH-mutant gliomas [58]; therefore, its assessment will be relevant in order to treat patients with appropriate targeted treatments.

## Conclusions

Our study confirms that liquid biopsy and cfDNA could be a complementary methods to tissue biopsy in the detection of IDH1 mutation, which can be used as a strong prognostic and predictive biomarker for a favorable clinical outcome in patients with glioma. The potential use of liquid biopsy opens an innovative field of circulating biomarkers research with a relevant impact on the characterization, prognosis, and clinical management of brain cancer.

## Data Availability

The data presented in this study are available in this article.

## Abbreviations

cfDNA: circulating free DNA
CSF: cerebrospinal fluid
EVs: extracellular vesicles
BBB: blood–brain barrier
WHO: World Health Organization
OS: Overall survival
GBM: glioblastoma
AF: Allelic Fraction
